# Validation of smartphone-based heart rate variability measurement against ECG in patients with depression

**DOI:** 10.1007/s00406-026-02239-5

**Published:** 2026-04-25

**Authors:** Lion D. Comfort, Mario Müller, Erich Seifritz, Sebastian Olbrich

**Affiliations:** Department of Adult Psychiatry and Psychotherapy, University Hospital of Psychiatry Zurich, Lenggstrasse 31, 8008 Zurich, Switzerland

**Keywords:** HRV, SPPG, Smartphone, Depression, Biomarker, ECG

## Abstract

Heart rate variability (HRV) describes time fluctuations between consecutive heart beats, providing insight into the sympathetic and parasympathetic branches of the autonomic nervous system. In depressed patients, HRV has shown reduction due to autonomic dysregulation. Traditionally, its measurement is conducted using electrocardiography (ECG). A novel approach is measurement through a conventional smartphone via photoplethysmography (SPPG), which has not yet been explored in depressed patients. Thus, we developed *PULSAR*, an SPPG application which measures the user’s pulse wave using the smartphone camera and flash, without the need for external devices, to calculate HRV. To validate *PULSAR*, simultaneous 5-minute resting-state ECG and SPPG measurements were conducted in 15 healthy individuals and 15 depressed patients. In terms of analytical validation, the SPPG-derived HRV parameters demonstrated high correlations to their ECG- derived counterparts while systematically overestimating most variables. This misestimation is attributed to physiological pulse wave characteristics, motion artifacts and the low sampling rate in SPPG measurements. Regarding clinical validation, HRV parameters in the depressed population were significantly reduced compared to healthy controls in both ECG and SPPG recordings. The frequency-domain parameter LF power, which primarily reflects sympathetic nervous system activity, emerged as the most robust variable for both clinical and analytical validation. Its SPPG-derived value showed a strong correlation with its ECG counterpart (*r* = 0.84) and a significant reduction in depressed patients (*p* = 0.021). Future iterations of the *PULSAR* application should include covariate adjustment, focus on addressing its overestimation of HRV parameters, implementing longitudinal measurements in depressed patients, and potentially incorporating biofeedback techniques to introduce a therapeutic dimension.

## Introduction

### Heart rate variability and depression

Depression is a common illnesses, with an estimated 5.7% of adults affected globally [1]. The average lifetime prevalence of major depressive disorder is projected to be around 15% in high-income countries [2]. While traditionally being diagnosed clinically and by exclusion, the search for appropriate cost-effective, easy-access diagnostic and predictive biomarkers is an ongoing and challenging task [3].

Heart Rate Variability (HRV) describes the time fluctuations in between consecutive heart beats. The heartbeat is regulated by the sinoatrial node, which in turn is influenced by the sympathetic and the parasympathetic nervous system (SNS and PNS) [4]. Thus, HRV offers a non-invasive insight into the autonomic nervous system (ANS) whose main branches are the SNS and PNS. A decrease in HRV is associated with a predominant SNS activity (“fight or flight”), whereas an elevated HRV indicates increased PNS activity (“rest and digest”) [5]. Different approaches of quantifying HRV have been established, whereas the time-domain and the frequency-domain parameters constitute the most common groups in addition to non-linear methods [4]. Various time-domain indexes quantify the amount of variability in inter-beat intervals and can be used to interpret both the general ANS and the specific PNS activity. For calculating the frequency-domain, the fluctuations of the heartbeat signal are assigned to different frequency bands using spectral analysis. The high frequency (HF) band can be used to draw conclusions about PNS activity, while the low frequency (LF) band is an indicator of both SNS and PNS activity, with a predominance of SNS influence [6, 7].

HRV is an active subject of research for psychiatry in general [8–10] and depression in particular [6, 11, 12]. It has been demonstrated that patients with major depression show multiple significantly reduced indexes of the time-domain as well as significantly reduced LF and HF parameters in the frequency-domain [6]. The effects of altered SNS and PNS activity in depressed patients can be explained through the neurovisceral integration model, according to which emotional dysregulation is defined by interactions between cerebral affective systems and the ANS [13]. Although antidepressant medication seems to play a role in influencing HRV [14], its decrease in depressed patients appears to be independent of pharmacological treatments [6, 15].

### Smartphone measurement of heart rate variability

The standard measurement method of obtaining HRV is electrocardiography (ECG) [4], which through high sampling rate and distinctive inter-beat intervals between R peaks allows for ideal measurement accuracy. An alternative, simpler way of detecting heart beats is photoplethysmography (PPG), which, in contrast, detects a pulse wave instead of an electrical signal [16]. The usage of pulse rate variability (PRV) has largely been applied as a validated surrogate of HRV but is not without challenges [17, 18]. In psychiatric research in particular, PPG derived HRV has shown high diagnostical potential [19]. The big advantage of PPG over ECG is its simplicity and widespread availability. Modern smartphones can turn into a PPG device by using the integrated flash as light source and the camera as photodetector [20, 21]. The validity of approximating HRV by the use of smartphone PPG (SPPG) has been suggested in small-scale studies with healthy individuals [22–24].

As previously stated, the topic of HRV in psychiatry is intensely researched, and today’s universal use of smartphones potentially offers a simple and cost-effective way of estimating a depressed patient’s HRV. Although suggested for psychophysiological research [25, 26], to this date, the promising idea of approximating a depressed patient’s HRV via SPPG alone has not yet been implemented. Thus, we developed *PULSAR*, an SPPG-based HRV application, and validated its performance in depressed patients and healthy individuals through simultaneous ECG-based HRV assessment.

In this study, it was hypothesized that.

1. HRV parameters derived from SPPG correlate with those derived from ECG (analytical validation).
2. SPPG-derived HRV parameters differ significantly between depressed patients and healthy individuals (clinical validation).

## Methods

### Design and participants

We conducted a comparative, observational, cross-sectional validity study with 15 depressed patients and 15 healthy individuals. Depressed subjects (mean age 35.5 years, SD = 12.8 years, 9 males, 6 females) were recruited from the hospital’s specialized wards for depressive disorders. Inclusion criteria were age 18–65 years and a moderate to severe depressive episode following the criteria of ICD-10 as confirmed by a senior psychiatrist, with severity levels additionally assessed by the Montgomery–Åsberg Depression Rating Scale (MADRS) [27]. MADRS is a ten-item questionnaire on depressive symptoms, assessed by a mental health professional. It yields a score from 0 to 60 and allows the classification of a depressive episode from mild to severe. Exclusion criteria were any additional currently untreated major psychiatric conditions, any current untreated/unstable non-psychiatric conditions, any forms of chronic cardiac arrhythmia or treatments interfering with ANS regulation of heartbeat. Healthy individuals (mean age = 35.7 years, SD = 13.6 years, 9 males, 6 females) were recruited from the private and professional network and matched precisely by age group and sex (See Table 1), being subjected to the same exclusion criteria.

**Table 1:** Demographic characteristics of the study sample–categorized by age and gender

Group	Number	Age	Males	Females
Depressed	15	< 27	2	2
		27–45	4	3
		> 45	3	1
Healthy	15	< 27	2	2
		27–45	4	3
		> 45	3	1

This study was approved by the local ethics committee (Ethikkomission Zürich, BASEC ID 2023 − 01273). All participants provided written informed consent prior to participation, and their privacy rights were observed throughout the study.

### Measurements

We conducted a short-term, 5-minute measurement of heart rate by ECG and smartphone simultaneously. Participants were resting in a 30° semi-recumbent position on an examination table and instructed to remain as immobile possible. A total of three consecutive measurements per participant was conducted to ascertain the measurement with the ideal conditions (absence of artifacts and noise in both measurement modalities).

#### ECG

For calculating ECG derived HRV, only the R peak intervals are relevant [28]. Therefore, we used the bipolar limb recordings as sufficient measurement modalities [29]. For ECG measurement we used Kalamed KEC-1000^®^, which recorded the electrical signal with a sampling rate of 500 Hz (Hz). ECG curves were plotted with the corresponding Kalamed ^®^ software and exported in the CSV file format. We then chose the ECG channels with the most prominent R peak for HRV analysis visually.

#### SPPG

**Fig. 1:**
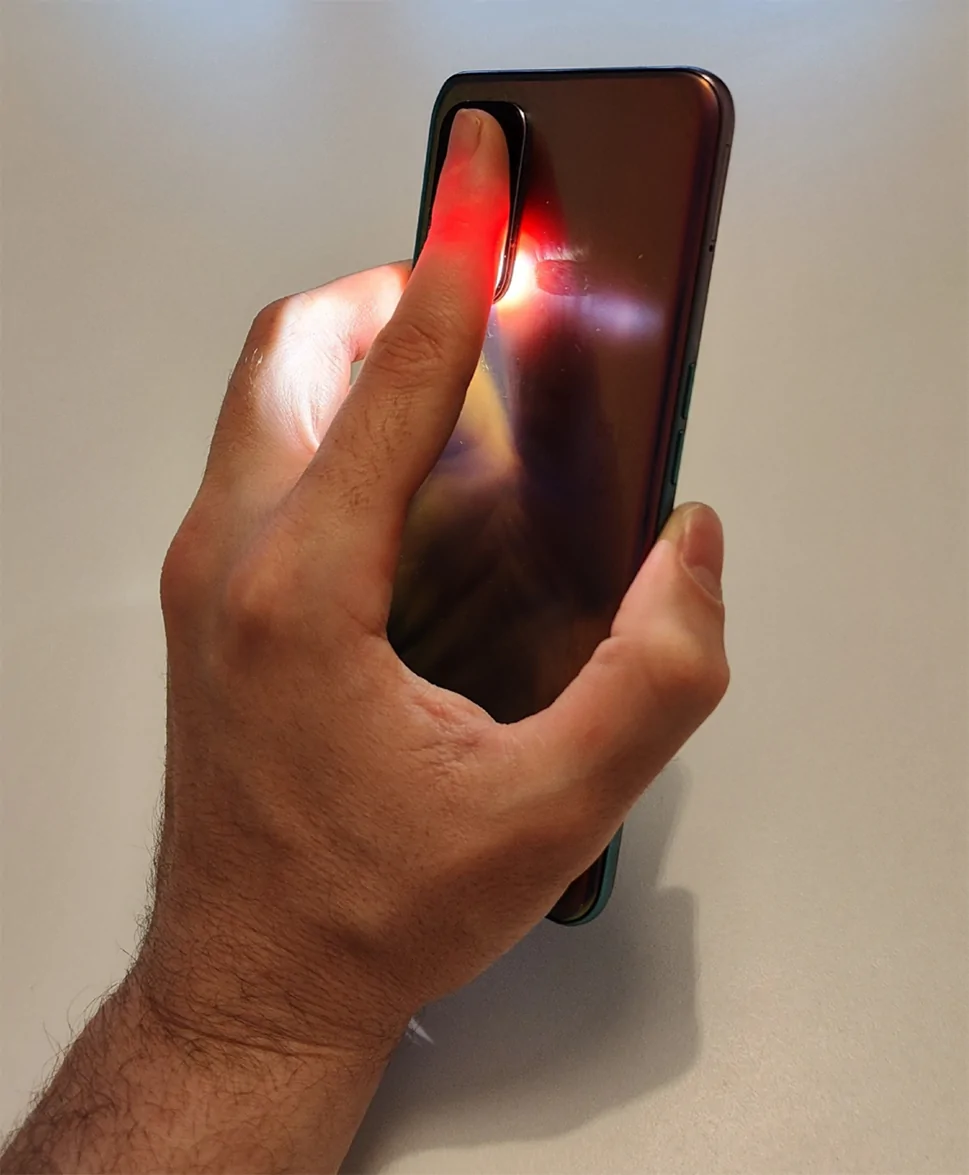
PULSAR in use. The user places his index finger on the smartphone camera while the flash illuminates the finger

For recording the SPPG curve, we developed the Android ^®^ application *PULSAR*. Upon launching the application, it assigns the participant a distinctive identifier and shows user instructions. The user’s index finger is placed firmly on the smartphone camera in a way that the finger fully covers the camera, and the smartphone flash illuminates the finger (see Fig. 1). During measurement, pulse peaks are detected in real-time and beats per minute are calculated and displayed. In addition, the user is given feedback on each detected pulse peak by slight vibration of the smartphone and a visual pulsating signal on the display. After the 5-minute measurement, HRV parameters are calculated (see *HRV Analysis* below) and displayed to the user and are ready for export for further analysis.

**Fig. 2:**
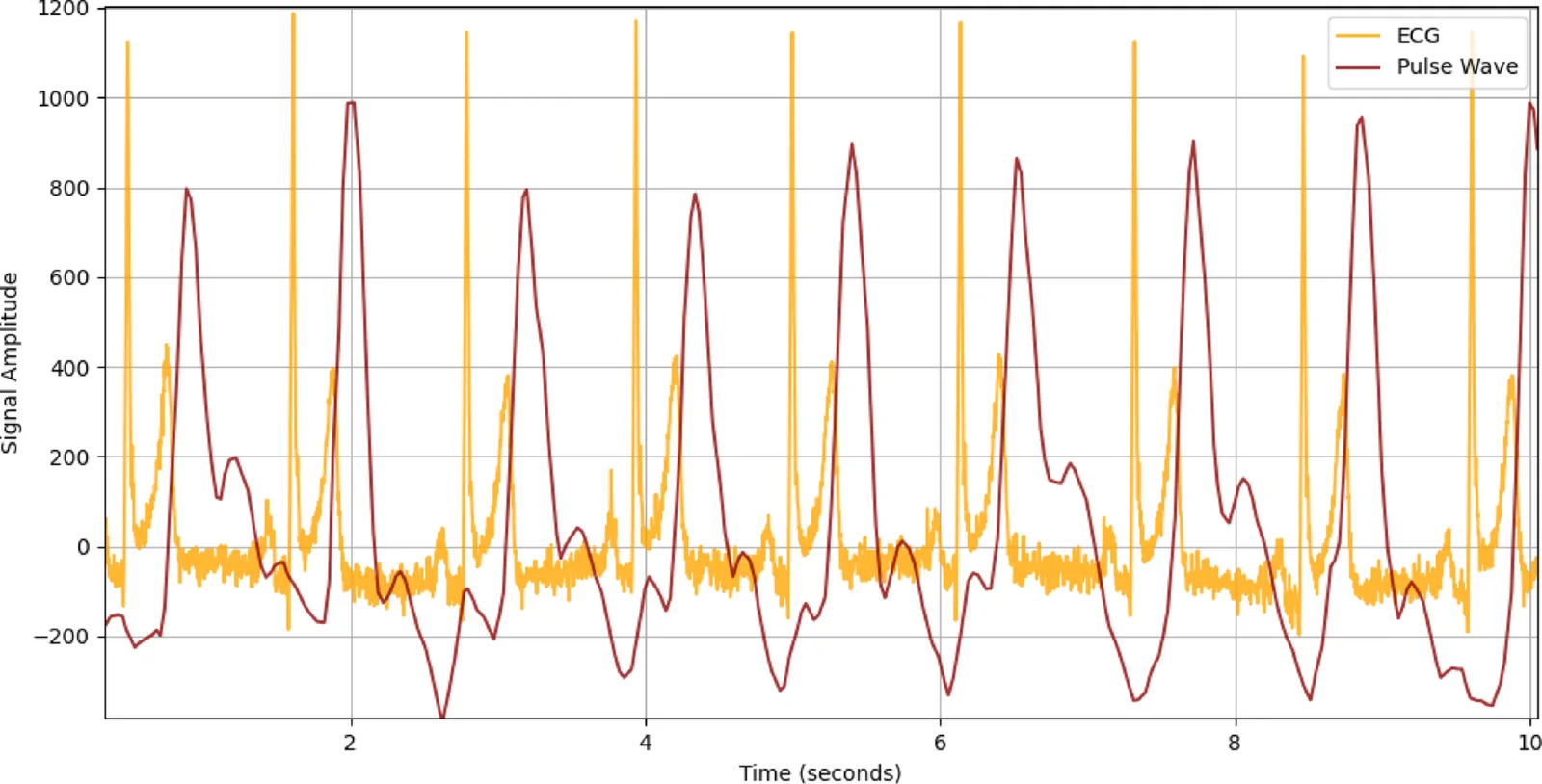
Exemplary PULSAR recording over 10 s (red) mapped over simultaneous ECG recording (orange)

*PULSAR* works by recording a video of the finger placed on the camera with a sampling rate of circa 30 Hz and adding up the amount of the red color in each video pixel (sum of red). Whenever a pulse wave passes the finger, the amount of red decreases due to the increased blood volume in some pixels, which results in a decrease of the sum of red in each video frame. Meanwhile, the smartphone flash illuminates the user’s finger and thereby accentuates the changes in red color. The SPPG pulse wave is therefore recorded by calculating the alterations of the color red over time (See Fig. 2) in the user’s index finger. Under ideal conditions, both the systolic pulse peaks and diastolic peaks can be distinctly recorded. The delay between the ECG’s R-peak (ventricular depolarization) and the pulse peak arises from the time required for the pulse wave to propagate from the heart to the fingertip. For pulse wave detection, we used pre-existing open-source code from Erdosi Balint’s *HeartBeat* project [30], which we adapted and expanded for the *PULSAR* application.

We used the same Android ^®^ phone (Realme C11 ^®^) for all measurements to ensure the same testing conditions for all participants. Recordings were performed using default camera exposure and illumination settings without manual parameter adjustments.

### HRV analysis

**Table 2:** An overview of all Heart Rate Variability (HRV) parameters [4, 28] used in the current study as calculated by HeartPy

Parameter	Unit	Description
*Time-domain measures*		
SDNN	ms	Standard deviation of all NN intervals
SDSD	ms	Standard deviation of differences between adjacent NN intervals.
RMSSD	ms	Root mean square of successive RR interval differences
PNN20	%	Percentage of successive RR intervals that differ by more than 20 ms
PNN50	%	Percentage of successive RR intervals that differ by more than 50 ms
*Non-linear measures*		
SD1	ms	Poincaré plot standard deviation perpendicular the line of identity
SD2	ms	Poincaré plot standard deviation along the line of identity
SD1/SD2	%	Ratio of SD1-to-SD2
*Frequency-domain measures*		
VLF	ms^2^	Absolute power of the very-low-frequency band (0.0033–0.04 Hz)
LF	ms^2^	Absolute power of the low-frequency band (0.04–0.15 Hz)
HF	ms^2^	Absolute power of the high-frequency band (0.15–0.4 Hz)
P_TOTAL	ms^2^	Variance of all NN intervals (0–0.4 Hz)
LF_PERC	%	Relative power of the low-frequency band (0.04–0.15 Hz)
HF_PERC	%	Relative power of the high-frequency band (0.15–0.4 Hz)
LF_NU	nu	LF power in normalized units: LF/(P_TOTAL - VLF) * 100
HF_NU	nu	HF power in normalized units: HF/(P_TOTAL - VLF) * 100

Analysis of HRV parameters (see Table 2) was conducted using *HeartPy* (Version 1.2) [31], an open-source *Python* library, previously validated for ECG and PPG assessment of HRV [32–34]. Initially, ECG curves were cropped to match the exact measurement timespan of the 5-minute SPPG measurement. ECG and SPPG curves were analyzed using the *process* function of *HeartPy*, using Welch’s method [35] to extract the frequency spectrum for the frequency-domain. For PPG signals, pulse peaks were used as fiducial points for pulse-to-pulse interval estimation. For ECG, R-peaks were detected and NN intervals derived. *HeartPy*’s standard bandpass filtering and artifact rejection algorithms were applied. In some cases, SPPG curves showed some baseline wandering [36], which posed a challenge to the calculation of peak-peak intervals. We applied *HeartPy*’s filtering function *remove_baseline_wander* to correct the affected curves. In rarer instances, SPPG pulse peak amplitudes were not distinctive enough to obtain an accurate peak-peak interval. To solve this, the *enhance_peaks* function of *HeartPy* was applied. No interpolation, upsampling, or exclusion of recording segments was performed.

### Statistical analysis

For comparison between ECG measurements and Smartphone-based PULSAR analysis: Descriptive statistics were calculated to summarize the distributional aspects of both ECG and smartphone data. For each assessed HRV variable, the mean, median, standard deviation, and interquartile range were computed. A paired t-test was conducted for each variable to identify statistically significant differences between ECG and smartphone measurements. P-values were reported to assess the significance of these differences. Pearson correlation coefficients were used to quantify the linear association between the two methods and results were visualized in a heatmap to provide an overview of potential discrepancies. To visually inspect the relationship between measurements from both methods, scatterplots were created for each variable.

To evaluate systematic deviations and biases between the two measurement methods, metric-specific Bland-Altman analysis was performed for all assessed HRV parameters (time-domain, frequency-domain, and non-linear measures). This method plotted the mean of the two measurements against their difference for each variable, providing visual assessment of parameter-specific agreement, systematic deviations, outliers and potential proportional bias or heteroscedasticity beyond correlation-based assessments. Additionally, a statistical bias analysis was conducted to compare mean biases between healthy and depressed groups using t-tests to determine whether observed biases differed significantly.

For method comparison analyses, paired-sample effect sizes (Cohen’s dz) were calculated to quantify the magnitude of systematic differences between ECG and smartphone measurements.

Comparison between patients with depression and healthy controls: To assess the validity of the smartphone method in distinguishing between healthy and depressed individuals and its agreement with ECG data, a comprehensive analysis was conducted. Descriptive statistics and mean comparisons were performed for each variable between healthy and depressed individuals, separately for each method (ECG and smartphone) to determine whether differences between the groups were statistically significant. To account for multiple comparisons, the Benjamin-Hochberg correction (FDR-BH) was applied, balancing Type-I and Type-II errors while maintaining statistical power. Given the high correlations between HRV parameters, a stricter Bonferroni correction would have been too conservative, potentially masking true group differences. FDH-BH retained the highest number of significant results, reinforcing the robustness of observed differences without inflating false positives. Z-scores were calculated for ECG and smartphone variables for standardized comparisons, and the results were illustrated using box-and-whisker plots for healthy and depressed individuals, providing a visual representation of group differences.

Finally, to evaluate the predictive accuracy of both methods in distinguishing between depressed and healthy individuals, receiver operating characteristic (ROC) analyses were performed. AUC values, sensitivity, and specificity were calculated for each HRV variable.

In our statistical analysis we omitted the VLF parameter since its measurement is discouraged in short-term (e.g. ≤ 5 min) analysis of HRV. The HF_NU parameter was excluded as it is mathematically complementary to the LF_NU parameter [4], resulting in identical correlations and providing no additional analytical value. RMSSD and SD1 likewise quantify redundant information [37]. Both parameters were nevertheless retained in the analysis, as SD1 is required for calculation of the SD1/SD2 ratio, and reporting both metrics facilitates comparability with existing HRV literature.

## Results

### Comparison of smartphone and ECG results

**Fig. 3:**
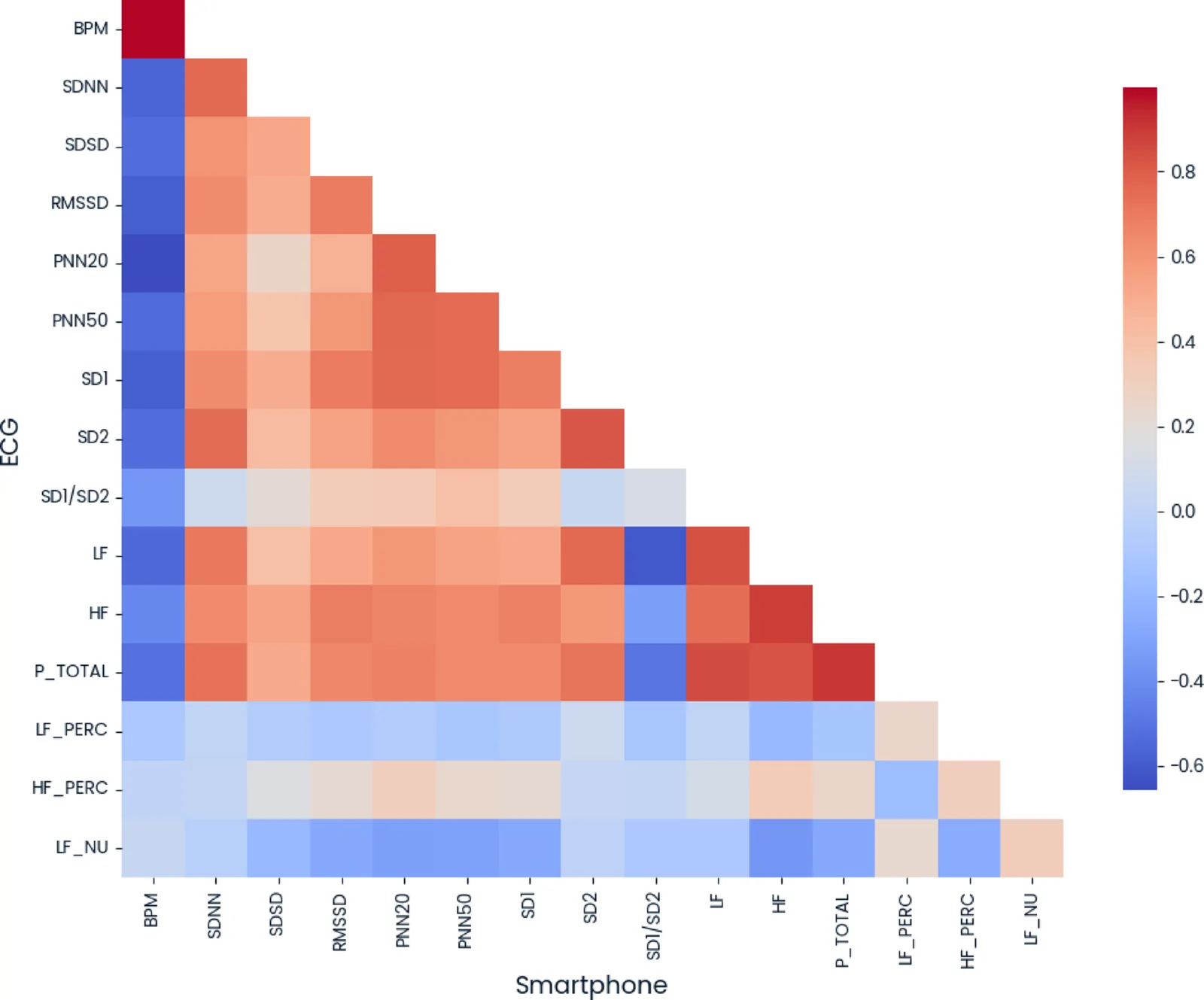
Heatmap of correlations between ECG- and smartphone-derived HRV parameters

Smartphone derived HRV values yielded higher mean values for most HRV parameters, exhibiting significant paired differences (see descriptive results in Table 3). Effect sizes (Cohen’s dz) indicated large systematic differences for most time-domain parameters, whereas LF showed only negligible effects. However, moderate to high correlations were found for most variables (e.g. BPM, P_TOTAL, LF), indicating a strong concordance between the two measurement methods (see heatmap in Fig. 3 for further details). In contrast, variables with lower associations, such as SD1/SD2 or LF_PERC, suggest greater variability between methods.

**Table 3:** Descriptive statistics of and associations between the ECG- and smartphone-derived HRV parameters

Variable	ECG Mean	ECG SD	ECG Median (IQR)	Smartphone Mean	Smartphone SD	Smartphone Median (IQR)	t-test *p*-value	Cohen’s dz	Correlation
BPM	74.94	13.44	69.87 (64.20; 84.53)	75.25	13.12	70.06 (64.90; 84.40)	0.095	−0.32	0.997
SDNN	46.75	21.88	41.57 (30.13; 67.06)	66.28	22.43	60.28 (47.90; 82.05)	0.000	−1.28	0.764
SDSD	23.85	17.58	19.31 (10.55; 28.41)	47.08	13.84	43.71 (34.58; 54.69)	0.000	−1.49	0.528
RMSSD	35.38	23.70	31.55 (16.36; 36.57)	67.42	18.38	64.45 (51.84; 80.86)	0.000	−1.87	0.695
PNN20	0.39	0.24	0.41 (0.17; 0.55)	0.75	0.06	0.75 (0.69; 0.79)	0.000	−1.79	0.791
PNN50	0.12	0.15	0.07 (0.01; 0.12)	0.37	0.11	0.35 (0.27; 0.44)	0.000	−2.42	0.757
SD1	25.01	16.75	22.31 (11.57; 25.85)	47.47	12.77	45.56 (36.66; 57.14)	0.000	−1.82	0.682
SD2	59.96	27.29	55.12 (41.08; 82.35)	69.40	29.52	63.64 (49.81; 85.39)	0.006	−0.54	0.817
SD1/SD2	0.41	0.16	0.36 (0.29; 0.51)	0.76	0.24	0.74 (0.60; 1.01)	0.000	−1.28	0.123
LF	852.88	783.81	465.33 (186.68; 1608.14)	935.12	727.58	736.28 (330.93; 1432.01)	0.302	−0.19	0.842
HF	601.92	797.59	279.64 (111.63; 813.59)	1243.06	992.14	1014.77 (542.54; 1539.74)	0.000	−1.41	0.893
P_TOTAL	1454.81	1469.87	793.00 (335.90; 2388.71)	2178.18	1587.28	1873.30 (1119.03; 2938.36)	0.000	−1.09	0.908
LF_PERC	37.63	11.94	39.09 (27.44; 47.15)	32.80	11.32	33.02 (24.05; 40.36)	0.072	0.34	0.261
HF_PERC	23.19	12.76	21.25 (13.41; 30.83)	46.23	14.05	47.25 (34.92; 56.82)	0.000	−1.46	0.309
LF_NU	62.76	15.80	66.48 (56.59; 73.80)	41.97	14.22	41.69 (28.10; 52.84)	0.000	1.18	0.314

**Fig. 4:**
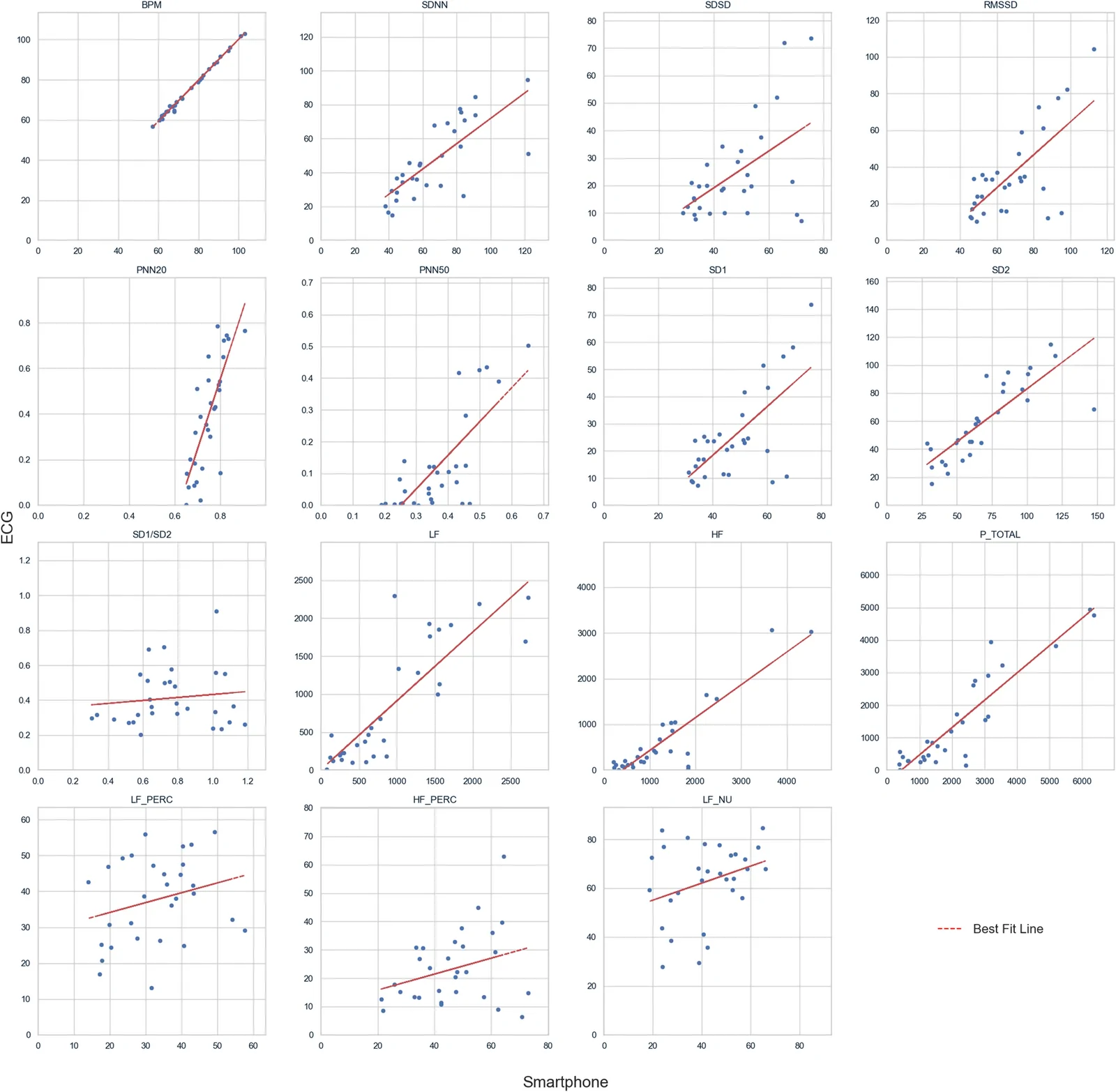
Scatterplots of ECG- and smartphone-derived HRV parameters with fitted regression lines

Accordingly, scatterplots in Fig. 4 demonstrate a clear alignment along the trendlines, indicating a strong overall agreement between the two methods. For variables such as BPM, LF, or P_TOTAL, data points closely follow the line of best fit, suggesting consistent measurement performance. Systematic deviations are observed in variables such as SDNN, SDSD, PNN20, or SD1, where smartphone measurements tend to be slightly higher at lower ECG values but converge and align at higher levels. In contrast, variables such as SD1/SD2 and LF_PERC exhibit a greater degree of scatter, indicating potential measurement variability while still suggesting an underlying agreement trend.

**Fig. 5:**
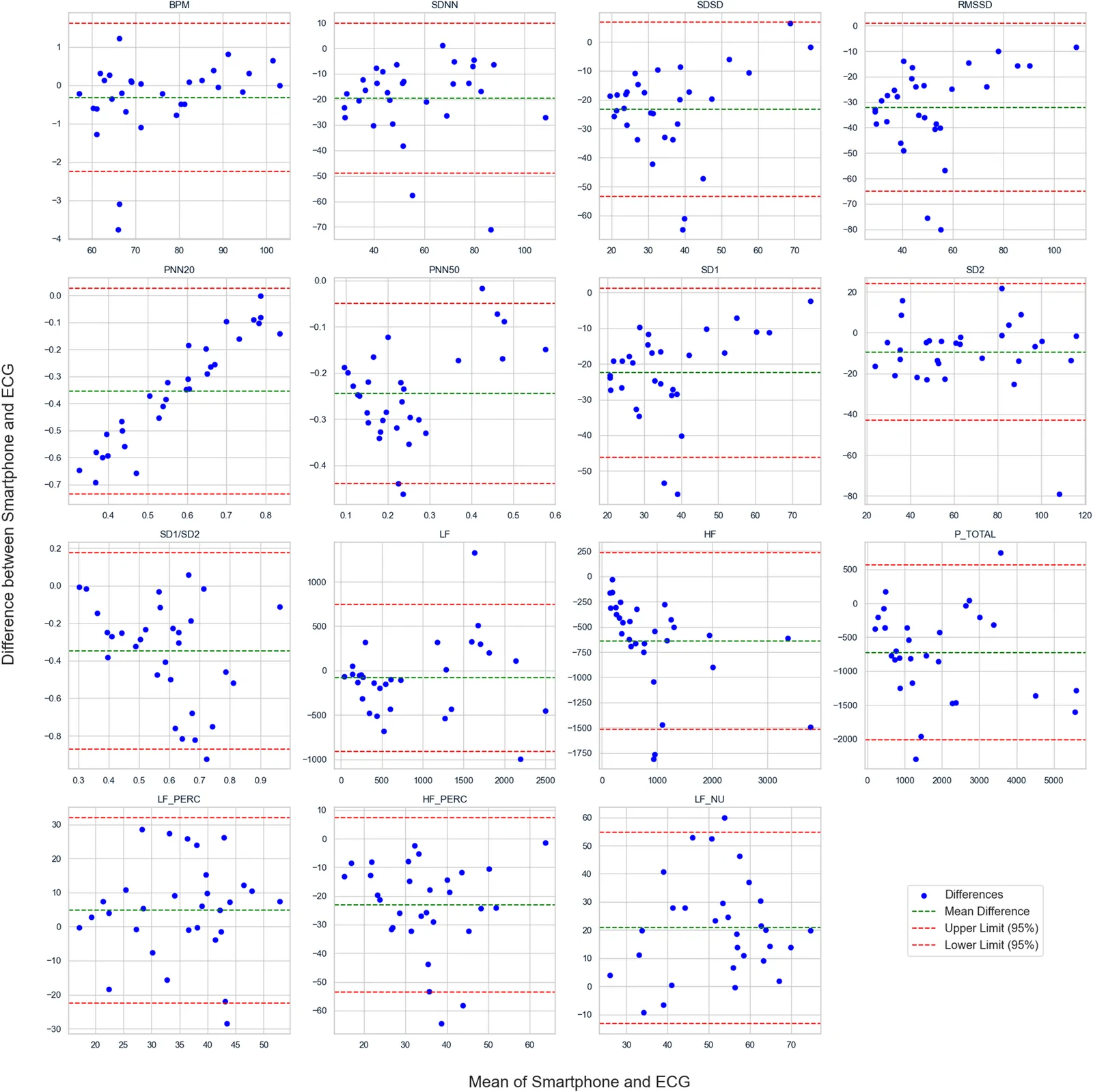
Bland-Altman plots analyzing the agreement between ECG- and smartphone-derived HRV measurements

Similarly, the Bland-Altman analyses in Fig. 5 demonstrate an overall strong agreement between the two methods for most variables. Variables such as BPM, SDNN, and SD2 show differences that are consistently centered close to the zero line, with only a few outliers outside the 95% limits of agreement.

In contrast, systematic deviations are observed for variables such as PNN20, RMSSD, and SDSD, where smartphone values tend to be higher at lower mean levels but align more closely with ECG measurements as the means increase. These patterns suggest a systematic (and thus predictable) shift, which further supports the trends identified in the scatterplots.

The bias analysis confirms a consistent level of agreement between the ECG and smartphone measurements across healthy and depressed groups for most variables (see Table 4). A clear exception is SD1/SD2, where a significant group-specific difference in bias suggests a systematic deviation. Additionally, SDSD and PNN20 variables are close to significance, suggesting trends toward group-specific bias that warrant further investigation.

For most variables, including BPM, SDNN, LF, HF, and P_TOTAL, the mean biases remain comparable across groups, underscoring the robustness of both measurement methods. These findings complement the Bland-Altman results, supporting the reliability of the smartphone as a viable alternative to ECG for most HRV parameters.

**Table 4:** Bias analysis comparing ECG- and smartphone-derived HRV parameters in healthy and depressed individuals

Variable	Healthy Mean Bias	Healthy SD	Depressed Mean Bias	Depressed SD	t-statistic	*p*-value
BPM	−0.59	1.27	−0.05	0.58	−1.51	0.143
SDNN	−17.42	17.05	−21.64	13.39	0.75	0.457
SDSD	−17.71	13.39	−28.76	16.14	2.04	0.051
RMSSD	−26.78	13.06	−37.3	19.47	1.74	0.093
PNN20	−0.29	0.15	−0.42	0.22	1.88	0.07
PNN50	−0.24	0.1	−0.25	0.1	0.1	0.924
SD1	−18.57	9.62	−26.34	13.77	1.79	0.084
SD2	−8.52	22.65	−10.35	10.33	0.28	0.778
SD1/SD2	−0.22	0.22	−0.47	0.27	2.77	0.01
LF	−31.02	568.95	−133.45	226.58	0.65	0.522
HF	−691.12	358.25	−591.16	542.71	−0.6	0.556
P_TOTAL	−722.13	651.14	−724.61	704.62	0.01	0.992
LF_PERC	0.91	13.58	8.75	14.05	−1.55	0.131
HF_PERC	−19.64	8.98	−26.45	20.28	1.19	0.244
LF_NU	17.26	11.54	24.34	22	−1.1	0.279

### Comparison of healthy and depressed population

**Fig. 6:**
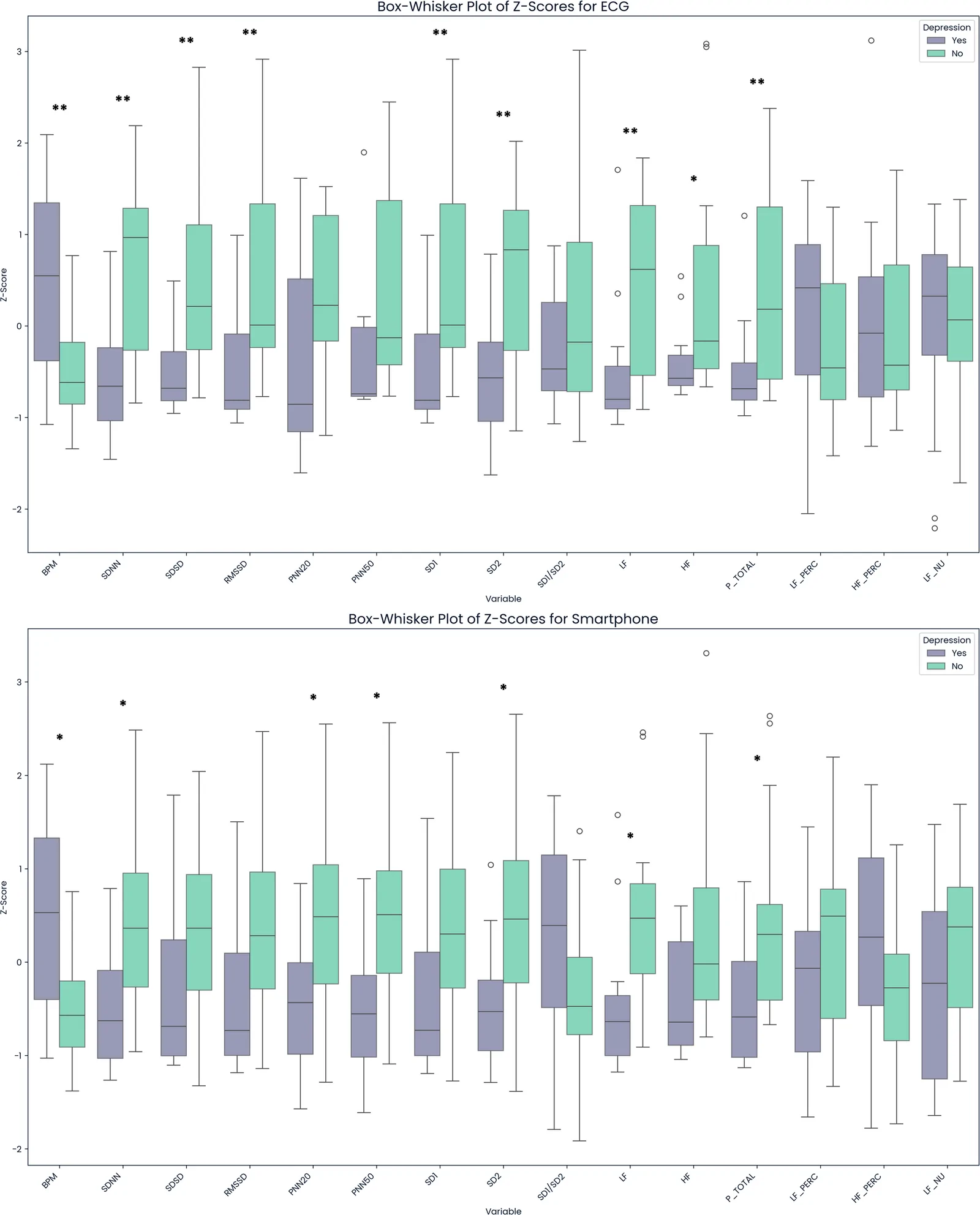
Box-and-whisker plot of Z-scores for ECG- and smartphone- derived HRV parameters, comparing depressed (blue) and healthy (green) individuals. * for *p* < 0.05; ** for *p* < 0.01

The results of the group comparisons indicate that ECG-based HRV variables effectively differentiate between depressed and healthy individuals, with smartphone measurements largely replicating these findings (See Table 5; Fig. 6a and b). To control for multiple comparisons, p-values were adjusted using the FDR-BH correction, ensuring a balanced approach between Type-I and Type-II errors. BPM and LF show strong agreement between methods, with similar means and consistent group differences, indicating their reliability in detecting depressive states. Systematic shifts were observed for SDNN, SD2, and P_TOTAL, where smartphone measurements tended to be higher than ECG values, yet group differences remained comparable between the two assessment methods. These differences remained statistically significant after correction, supporting the robustness of these HRV markers. In contrast, RMSSD, HF, and PNN20 did not remain significant after correction, indicating limited discriminatory power.

**Table 5:** Descriptive statistics of and comparison of HRV parameters between healthy and depressed individuals for both ECG- and smartphone-derived measurements

Variable	ECG					Smartphone				
	Mean Healthy ± SD	Mean Depressed ± SD	Difference	*p*-value	Adj. *p*-value (FDR-BH)	Mean Healthy ± SD	Mean Depressed ± SD	Difference	*p*-value	Adj. *p*-value (FDR-BH)
BPM	68.23 ± 8.20	81.65 ± 14.51	−13.42	0.004	**0.008**	68.82 ± 8.09	81.69 ± 14.21	−12.88	0.005	**0.021**
SDNN	59.52 ± 21.00	33.98 ± 14.19	25.54	0.001	**0.008**	76.94 ± 23.40	55.62 ± 15.86	21.32	0.007	**0.021**
SDSD	33.28 ± 20.06	14.43 ± 6.88	18.85	0.002	**0.008**	50.99 ± 13.66	43.18 ± 13.32	7.80	0.125	0.144
RMSSD	47.34 ± 26.06	23.42 ± 13.32	23.92	0.004	**0.008**	74.12 ± 19.03	60.72 ± 15.55	13.40	0.044	0.072
PNN20	0.49 ± 0.21	0.30 ± 0.25	0.18	0.037	0.056	0.77 ± 0.06	0.72 ± 0.05	0.05	0.017	**0.036**
PNN50	0.18 ± 0.18	0.07 ± 0.11	0.11	0.053	0.072	0.42 ± 0.11	0.32 ± 0.09	0.11	0.007	**0.021**
SD1	33.47 ± 18.43	16.56 ± 9.42	16.91	0.004	**0.008**	52.04 ± 13.14	42.89 ± 10.98	9.15	0.048	0.072
SD2	75.35 ± 26.18	44.57 ± 18.70	30.78	0.001	**0.008**	83.87 ± 31.42	54.92 ± 19.22	28.95	0.005	**0.021**
SD1/SD2	0.45 ± 0.20	0.37 ± 0.11	0.08	0.172	0.215	0.68 ± 0.20	0.84 ± 0.25	−0.16	0.056	0.076
LF	1249.94 ± 788.39	455.83 ± 559.66	794.10	0.004	**0.008**	1280.95 ± 727.36	589.28 ± 557.85	691.67	0.007	**0.021**
HF	934.90 ± 993.73	268.94 ± 304.39	665.96	0.019	**0.032**	1626.02 ± 1165.27	860.11 ± 605.71	765.91	0.032	0.060
P_TOTAL	2184.84 ± 1636.92	724.78 ± 808.59	1460.06	0.004	**0.008**	2906.97 ± 1736.22	1449.39 ± 1032.60	1457.59	0.009	**0.023**
LF_PERC	35.65 ± 10.04	39.61 ± 13.64	−3.97	0.372	0.429	34.74 ± 12.43	30.86 ± 10.15	3.87	0.358	0.358
HF_PERC	22.40 ± 11.55	23.97 ± 14.23	−1.57	0.743	0.796	42.04 ± 12.18	50.42 ± 14.94	−8.38	0.103	0.129
LF_NU	62.39 ± 14.50	63.14 ± 17.51	−0.75	0.899	0.899	45.13 ± 13.53	38.80 ± 14.65	6.33	0.229	0.245

Building on these findings, we assessed the predictive accuracy of both methods by analyzing AUC values, sensitivity, and specificity for each variable. (See Table 6). Overall, AUC analysis shows high AUC values and moderate- to high sensitivity and specificity across time- and frequency domain HRV variables in both ECG and smartphone measurements, suggesting a strong discriminatory power for distinguishing between our healthy and depressed population. However, some variables, such as LF_PERC, HF_PERC and LF_NU showed inconsistent results. While the AUC values for these metrics are moderate in some cases, their overall discriminative ability (and thus clinical utility) remains limited due to minimal and statistically non-significant group differences observed earlier.

Notably, SD1/SD2 emerges as a variable where smartphone measurements outperform ECG by means of group differentiation, underscoring the smartphone’s potential competitive performance for selected metrics.

For BPM, the AUC, sensitivity and specificity were identical across ECG and smartphone recordings. This reflects the almost perfect rank correlation between both measurements (Spearman ρ = 0.99, *p* < 0.001; not tabulated), resulting in virtually identical participant ordering and therefore identical ROC characteristics.

**Table 6:** Area under the curve (AUC), sensitivity and specificity values of ECG- and smartphone-derived HRV parameters for distinguishing between healthy and depressed individuals

Variable	ECG			Smartphone		
	AUC	Sensitivity	Specificity	AUC	Sensitivity	Specificity
BPM	0.78	0.6	0.93	0.78	0.6	0.93
SDNN	0.84	0.8	0.73	0.8	0.73	0.8
SDSD	0.86	0.93	0.67	0.66	0.6	0.8
RMSSD	0.83	0.53	1	0.7	0.6	0.8
PNN20	0.71	0.53	0.93	0.76	0.8	0.73
PNN50	0.74	0.67	0.8	0.76	0.8	0.73
SD1	0.83	0.53	1	0.69	0.6	0.8
SD2	0.83	0.8	0.73	0.8	0.8	0.73
SD1/SD2	0.59	1	0.27	0.7	0.6	0.87
LF	0.8	0.87	0.67	0.81	0.87	0.8
HF	0.76	0.6	0.87	0.73	0.53	0.93
P_TOTAL	0.79	0.87	0.67	0.78	0.47	1
LF_PERC	0.61	0.6	0.73	0.59	0.8	0.53
HF_PERC	0.5	0.6	0.6	0.69	0.67	0.8
LF_NU	0.55	0.67	0.6	0.64	0.33	0.93

## Discussion

This study presents a novel modality for approximating reduced HRV in depressed patients using only a standard smartphone without external devices, validating it against the state-of-the-art assessment of HRV via ECG. We confirmed Hypothesis 1 by demonstrating that SPPG-derived HRV parameters are highly correlated to their ECG based counterparts during simultaneous 5-minute resting measurements in patients with depression and healthy individuals. Additionally, our depressed population exhibited a significant reduction in HRV parameters across time- and frequency domain measures in both ECG and SPPG recordings compared to the healthy group, confirming Hypothesis 2. While previous studies have either extensively examined altered HRV in depression or the usage of (S)PPG as a surrogate of the gold standard ECG, to the author’s knowledge, this is the first application of a smartphone-only approach in clinically depressed patients.

When using PPG-derived pulse signals as a surrogate for HRV, it is important to consider the fundamental physiological differences between cardiac electrical activity and the peripheral pulse waveform [17, 18]. PPG yields a smooth signal, and its fiducial point detection proves an important challenge. Moreover, peripheral pulse is subjected to autonomic tone and peripheral perfusion. Thus, pulse rate variability (PRV) can be influenced by numerous physiological influences, such as pulse transit time variability, vascular characteristics (e.g. vascular tone or influence of medication), baroreflex influences, site of PPG measurement and ectopic or arrhythmic events. These physiological considerations underscore that PRV should be interpreted as a related but not identical construct to HRV.

Previous studies, however, have demonstrated strong correlations between ECG- and SPPG derived HRV parameters across time- and frequency domain measures [22, 36, 38]. Regarding analytical validation, our findings replicated these results for BPM and the time- and frequency domain parameters with one exception in each case. However, SPPG measurement tended to overestimate most HRV variables, particularly at lower values, leading to significant differences between both measurement methods. An exception to this trend was the LF power parameter, which exhibited both a high correlation and a non-significant difference between SPPG and ECG. The robustness of this parameter was confirmed by additional analysis (Bland-Altman and bias analysis). We partly attribute this systematic overestimation to motion artifacts during our SPPG measurements. Involuntary finger movement during (S)PPG measurement can introduce gaps and falsely detected pulse peaks, significantly altering HRV results [38].

*PULSAR* has a relatively low sampling rate of 30 Hz, while established HRV literature recommends substantially higher sampling rates (≥ 250 Hz) for accurate interbeat discrimination [4], especially for frequency-sensitive parameters (e.g. RMSSD or HF). Previous studies have shown a systematic overestimation of PPG-derived HRV parameters at lower sampling rates, with the magnitude of overestimation increasing as the sampling rate decreases [39, 40]. This effect is attributed to a decrease in distinction of the pulse peak in lower sampling rates, leading to reduced precision in quantifying the interbeat intervals [39]. Modern smartphones video cameras can reach a sampling rate of up to 240 Hz, which can provide higher temporal precision for pulse interval extraction [41]. Moreover, upscaling is possible through interpolation techniques (e.g. parabola interpolation, cubic-spline, and linear regression methods) and can be recommended for PPG sampling below 32 Hz [42]. However, those modalities do not increase the intrinsic temporal resolution of the recorded signal. To accurately quantify HRV parameters using *PULSAR*, interpolation and calibration algorithms to correct for overestimation need to be implemented in the application. Nevertheless, current results remain sufficiently reliable for intra- and interindividual comparison of SPPG derived HRV parameters.

Recent systematic reviews and meta-analyses have consistently shown significant increases in BPM and decreases in HRV parameters across time- and frequency domain measures in depressed patients [6, 11, 39]. In terms of clinical validation, our study fully corroborates these findings through ECG-derived parameters and validates them for SPPG variables except for SDSD. This not only confirms previous research highlighting reduced HRV in depression but more importantly underscores the potential of SPPG in measuring said reduced HRV. Notably, the LF power parameter stood out again due to its strong performance: It exhibited a significant reduction in descriptive group comparisons (*p* = 0.021), a narrow interquartile range with minimal overlap in the box-and-whisker plots, and robust diagnostic accuracy in the ROC analysis for detecting depression (AUC = 0.81, sensitivity = 0.87, specificity = 0.80). Similarly, BPM demonstrated identical ROC characteristics across ECG and smartphone recordings. This reflects the very high rank concordance between both measurement modalities, indicating that discriminative performance is preserved despite minor systematic differences in absolute values. In contrast, non-linear parameters (SD1, SD2) as well as LF_PERC, HF_PERC and LF_NU showed limited discriminative relevance in our sample, which is consistent with the comparatively minor emphasis placed on these parameters in the existing literature.

These findings prompted a closer examination of the frequency domain of HRV. ANS influences lead to a fluctuating heart rate signal over time. Through spectral analysis, these oscillatory components can be broken down into different frequency bands [43], with the LF band being set at 0.03–0.15 Hz and the HF band at 0.15–0.4 Hz [44]. The LF band is proposed to be influenced by both PNS and SNS activity, whereas SNS activation appears to play a dominant role in its increase. The HF band is considered to be solely influenced by PNS activity [7]. In our depressed population, both LF and HF power parameters showed significant decreases, which suggests reduced activity of SNS as well as PNS branches of the ANS in depressed patients. Given LF power’s robustness as the most reliable variable measured by the *PULSAR* application, SPPG-derived measurements of this parameter can be proposed as a potential biomarker for gaining meaningful insights into the PNS and SNS activity of depressed individuals.

HRV has been suggested as a viable diagnostic biomarker for mental disorders in general [45–47] and depression in particular [11, 48, 49]. Patients with recent suicide attempts have exhibited significant reduction in HRV parameters across time- and frequency domain measures [50]. Beyond its diagnostic utility, heart rate analysis has demonstrated predictive potential for antidepressant medication with serotonin-norepinephrine reuptake inhibitors (SNRIs) [51]. Furthermore, a recent study utilized HRV parameters to discriminate between depressive responders and non-responders to ketamine therapy, underscoring its potential as a predictive biomarker for treatment outcomes [52]. Given that SPPG measurement of HRV offers a non-invasive, easy-access and cost-effective modality [21], its potential in detecting, stratifying and analysis of the predictiveness of HRV-derived biomarkers merits significant consideration.

### Strengths and limitations

We investigated a total of 14 common HRV parameters in addition to beats per minute (BPM) and conducted rigorous descriptive and visual analyses. This allowed us to perform a broad analytical and clinical validation process and ascertain the specific SPPG derived HRV parameters with the highest (e.g. LF power) and the least (e.g. SDSD) potential.

For this study, we used a relatively small dataset of *n* = 30 participants. While this sample size was deemed appropriate for an initial feasibility study in this research area, future studies should include a larger cohort to strengthen our findings.

Several important covariates known to influence BPM and HRV, including antidepressant medication, age, sex, physical fitness, smoking and time of the day [53] were not systematically accounted for in this study. Owing to the feasibility-oriented design and limited sample size, covariate adjustment or subgroup analyses were not deemed statistically justified. Future studies with larger cohorts should address these covariates in line with current HRV reporting guidelines.

Skin pigmentation was not systematically assessed in this study and represents an important limitation. Increased melanin concentration alters light absorption and reflectance properties of the skin, which can reduce PPG signal amplitude and signal-to-noise ratio [54]. This may introduce systematic measurement differences or increased data loss in individuals with darker skin tones, thereby limiting the generalizability of the present findings. Given the feasibility-driven design and limited sample size, no participant skin tone information (e.g. Fitzpatrick scale or objective melanin index) was collected. Future studies should explicitly assess skin pigmentation and evaluate its influence on SPPG-derived HRV metrics and should consider mitigation strategies such as wavelength selection or algorithmic compensation to improve equity and external validity of measurements.

Identical measurement conditions (same smartphone, reclining position, finger placement, room lighting) were maintained for all participants. In practical applications, however, *PULSAR* would be used in a variety of settings where external factors, particularly lighting conditions, could affect measurement precision.

The study did not include complementary agreement statistics (e.g. ICC or error-in-variables regression models), which may further refine reliability estimates in future larger cohorts.

Physiological contributors to PRV–HRV divergence were not explicitly modeled and represent an important limitation of this feasibility study. Additionally, *PULSAR’s* SPPG measurements seem to be susceptible to movement artifacts, which can distort the recorded pulse wave and the derived HRV parameters. To mitigate this, we selected the result least affected by artifacts from three measurements and applied *HeartPy’s* filtering functions. For future iterations, we aim to implement detection and correction algorithms into *PULSAR* to automatically reduce movement artifacts. Moreover, *PULSAR’s* relatively low sampling rate of 30 Hz presents a limitation to measurement accuracy. Future iterations should aim for higher sampling rate for acquisition of SPPG signal and additionally implement interpolation algorithms for signal upscaling.

The study did not include additional comparison with a clinical-grade PPG sensor, which limits the ability to disentangle smartphone-specific measurement effects from fundamental physiological differences between PPG and ECG derived HRV. Moreover, in our current study, no supplementary SSPG signal and waveform characteristics (such as pulse amplitude, pulse arrival time, pulse shape indices, perfusion-related measures and quality metrics) were systematically analyzed or reported. Future multimodal research incorporating ECG, SPPG, and dedicated PPG sensors as well as incorporation of PPG signal waveform characteristics and quality indicators may enable a more precise characterization of sensor- versus physiology-driven discrepancies.

Future research should focus on longitudinally assessing HRV parameters in depressed patients using *PULSAR*, with particular emphasis on the LF power parameter. Such studies would not only reinforce our findings but also provide patients with meaningful insights into their ANS by offering a user-friendly, HRV-derived score. Numerous studies have demonstrated the efficacy of HRV biofeedback training [55, 56]. Future iterations of our smartphone application could integrate biofeedback techniques, such as breathing exercises [57], adding a therapeutic dimension to *PULSAR*.

## Conclusion

In terms of analytical validation, our study demonstrated strong to very strong correlations between most SPPG-derived HRV parameters and their ECG counterparts. However, SPPG measurements exhibited systematic overestimations, particularly at lower HRV values. We attribute this to physiological PRV-HRV divergence, motion artifacts and reduced sampling rate. Regarding clinical validation, our depressed population revealed a significant reduction in HRV measured by SPPG- and confirmed by ECG-based measurements. Among the assessed variables, the frequency-domain LF power parameter (*r* = 0.84, *p* = 0.021, AUC = 0.81, sensitivity = 0.87, specificity = 0.80), which is chiefly dependent on sympathetic nervous system activity, emerged as the most reliable metric, demonstrating strong potential for detecting HRV reductions in depressed patients. These findings support the theory of autonomic dysregulation with altered sympathetic and reduced parasympathetic nervous system activity in depression.

In conclusion, our study demonstrated the feasibility of approximating HRV reduction in depression using SPPG alone, without the need for external devices. This further reinforces the role of HRV as a biomarker in depression and highlights smartphone-based measurement as an accessible and cost-effective modality for its acquisition. Future studies should include covariate adjustment, honor the impact of skin pigmentation, aim to address the systematic overestimations observed in SPPG measurements (e.g. signal upscaling, additional PPG sensors), conduct longitudinal assessment of HRV in larger groups of depressed patients and incorporate biofeedback techniques into our smartphone application.

## Data Availability

Data will be made available upon reasonable request to the corresponding author.

## References

[1] WHO (2025) WHO Fact sheet depressive disorder. https://www.who.int/news-room/fact-sheets/detail/depression. Accessed 15 Nov 2025

[2] Bromet E, Andrade LH, Hwang I, Sampson NA, Alonso J, de Girolamo G et al (2011) Cross-national epidemiology of DSM-IV major depressive episode. BMC Med 9:90. 10.1186/1741-7015-9-90 PMC3163615 21791035

[3] Arns M, van Dijk H, Luykx JJ, van Wingen G, Olbrich S (2022) Stratified psychiatry: tomorrow’s precision psychiatry? Eur Neuropsychopharmacol 55:14–9. 10.1016/j.euroneuro.2021.10.863 34768212

[4] Marek M (1996) Heart rate variability. Standards of measurement, physiological interpretation, and clinical use. Task Force of the European Society of Cardiology and the North American Society of Pacing and Electrophysiology. Eur Heart J 17(3):354–81. 10.1161/01.CIR.93.5.1043 8737210

[5] Siepmann M, Weidner K, Petrowski K, Siepmann T (2022) Heart Rate Variability: a measure of cardiovascular health and possible therapeutic target in dysautonomic mental and neurological disorders. Appl Psychophysiol Biofeedback 47(4):273–287. 10.1007/s10484-022-09572-0 PMC9718704 36417141

[6] Koch C, Wilhelm M, Salzmann S, Rief W, Euteneuer F (2019) A meta-analysis of heart rate variability in major depression. Psychol Med 49(12):1948–1957. 10.1017/S0033291719001351 31239003

[7] Sgoifo A, Carnevali L, Alfonso ML, Amore M (2015) Autonomic dysfunction and heart rate variability in depression. Stress 18(3):343–352. 10.3109/10253890.2015.1045868 26004818

[8] Alvares GA, Quintana DS, Hickie IB, Guastella AJ (2016) Autonomic nervous system dysfunction in psychiatric disorders and the impact of psychotropic medications: a systematic review and meta-analysis. J Psychiatry Neurosci 41(2):89–104. 10.1503/jpn.140217 PMC4764485 26447819

[9] Wang Z, Luo Y, Zhang Y, Chen L, Zou Y, Xiao J et al (2023) Heart rate variability in generalized anxiety disorder, major depressive disorder and panic disorder: a network meta-analysis and systematic review. J Affect Disord 330:259–66. 10.1016/j.jad.2023.03.018 36914118

[10] Quintana DS, Alvares GA, Heathers JA (2016) Guidelines for reporting articles on psychiatry and heart rate variability (GRAPH): recommendations to advance research communication. Transl Psychiatry 6(5):e803. 10.1038/tp.2016.73 PMC5070064 27163204

[11] Schiweck C, Piette D, Berckmans D, Claes S, Vrieze E (2019) Heart rate and high frequency heart rate variability during stress as biomarker for clinical depression. A systematic review. Psychol Med 49(2):200–211. 10.1017/S0033291718001988 30134999

[12] Kemp AH, Quintana DS, Gray MA, Felmingham KL, Brown K, Gatt JM (2010) Impact of depression and antidepressant treatment on heart rate variability: a review and meta-analysis. Biol Psychiatry 67(11):1067–1074. 10.1016/j.biopsych.2009.12.012 20138254

[13] Thayer JF, Lane RD (2000) A model of neurovisceral integration in emotion regulation and dysregulation. J Affect Disord 61(3):201–. 10.1016/s0165-0327(00)00338-4. 16 11163422

[14] Licht CM, de Geus EJ, van Dyck R, Penninx BW (2010) Longitudinal evidence for unfavorable effects of antidepressants on heart rate variability. Biol Psychiatry 68(9):861–8. 10.1016/j.biopsych.2010.06.032 20843507

[15] Brown L, Karmakar C, Gray R, Jindal R, Lim T, Bryant C (2018) Heart rate variability alterations in late life depression: a meta-analysis. J Affect Disord 235:456–466. 10.1016/j.jad.2018.04.071 29679898

[16] Allen J (2007) Photoplethysmography and its application in clinical physiological measurement. Physiol Meas 28(3):R1–39. 10.1088/0967-3334/28/3/R01 17322588

[17] Schäfer A, Vagedes J (2013) How accurate is pulse rate variability as an estimate of heart rate variability? A review on studies comparing photoplethysmographic technology with an electrocardiogram. Int J Cardiol 166(1):15–29. 10.1016/j.ijcard.2012.03.119 22809539

[18] Mejía-Mejía E, May JM, Torres R, Kyriacou PA (2020) Pulse rate variability in cardiovascular health: a review on its applications and relationship with heart rate variability. Physiol Meas 41(7):07TR1. 10.1088/1361-6579/ab998c 32498055

[19] Lyzwinski LN, Elgendi M, Menon C (2023) The Use of Photoplethysmography in the Assessment of Mental Health: Scoping Review. JMIR Ment Health 10:e40163. 10.2196/40163 PMC10262030 37247209

[20] Jonathan E, Leahy M (2010) Investigating a smartphone imaging unit for photoplethysmography. Physiol Meas 31(11):N79–83. 10.1088/0967-3334/31/11/N01 20871134

[21] De Ridder B, Van Rompaey B, Kampen JK, Haine S, Dilles T (2018) Smartphone Apps Using Photoplethysmography for Heart Rate Monitoring: Meta-Analysis. JMIR Cardio 2(1):e4. 10.2196/cardio.8802 PMC6834218 31758768

[22] Vondrasek JD, Riemann BL, Grosicki GJ, Flatt AA (2023) Validity and efficacy of the Elite HRV smartphone application during slow-paced breathing. Sensors. 10.3390/s23239496 PMC10708620 38067869

[23] Holmes CJ, Fedewa MV, Winchester LJ, MacDonald HV, Wind SA, Esco MR (2020) Validity of smartphone heart rate variability pre- and post-resistance exercise. Sensors (Basel). 10.3390/s20205738 PMC7600564 33050249

[24] Peng RC, Zhou XL, Lin WH, Zhang YT (2015) Extraction of heart rate variability from smartphone photoplethysmograms. Comput Math Methods Med 2015:516826. 10.1155/2015/516826 PMC4309304 25685174

[25] Heathers JA (2013) Smartphone-enabled pulse rate variability: an alternative methodology for the collection of heart rate variability in psychophysiological research. Int J Psychophysiol 89(3):297–304. 10.1016/j.ijpsycho.2013.05.017 23751411

[26] Liu I, Ni S, Peng K (2020) Happiness at Your Fingertips: Assessing Mental Health with Smartphone Photoplethysmogram-Based Heart Rate Variability Analysis. Telemed J E Health 26(12):1483–1491. 10.1089/tmj.2019.0283 32101084

[27] Montgomery SA, Asberg M (1979) A new depression scale designed to be sensitive to change. Br J Psychiatry 134:382–389. 10.1192/bjp.134.4.382 444788

[28] Shaffer F, Ginsberg JP (2017) An overview of heart rate variability metrics and norms. Front Public Health 5:258. 10.3389/fpubh.2017.00258 PMC5624990 29034226

[29] Meek S, Morris F (2002) ABC of clinical electrocardiography.Introduction. I-Leads, rate, rhythm, and cardiac axis. BMJ 324(7334):415–418. 10.1136/bmj.324.7334.415 PMC1122339 11850377

[30] Balint E (2021) HeartBeat. Version 1.3. GitHub. https://github.com/berdosi/HeartBeat. Accessed 15 Nov 2025

[31] van Gent P (2018) HeartPy. Version 1.2. Read The Docs. https://python-heart-rate-analysis-toolkit.readthedocs.io/. Accessed 15 Nov 2025

[32] van Gent P, Farah H, Nes N, van Arem B (2018) Heart rate analysis for human factors: development and validation of an open source toolkit for noisy naturalistic heart rate data. semantic scholar. https://api.semanticscholar.org/CorpusID:70042780. Accessed 15 Nov 2025

[33] van Gent P, Farah H, van Nes N, van Arem B (2019) Analysing noisy driver physiology real-time using off-the-shelf sensors: heart rate analysis software from the taking the fast lane project. J Open Res Softw 7(2). 10.5334/jors.241

[34] Vićentić T, Rašljić Rafajilović M, Ilić SD, Koteska B, Madevska Bogdanova A, Pašti IA et al (2022) Laser-induced graphene for heartbeat monitoring with HeartPy analysis. Sensors. 10.3390/s22176326 PMC9460202 36080785

[35] Welch PD (1967) The use of fast Fourier transform for the estimation of power spectra: a method based on time averaging over short, modified periodograms. IEEE Trans Audio Electroacoust. 10.1109/TAU.1967.1161901

[36] Zhao H, Li T, Yang J, Pang C (2023) An error-bounded median filter for correcting ECG baseline wander. Health Inf Sci Syst 11(1):45. 10.1007/s13755-023-00235-w PMC10522562 37771394

[37] Ciccone AB, Siedlik JA, Wecht JM, Deckert JA, Nguyen ND, Weir JP (2017) Reminder: RMSSD and SD1 are identical heart rate variability metrics. Muscle Nerve 56(4):674–678. 10.1002/mus.25573 28073153

[38] Królak A, Wiktorski T, Bjørkavoll-Bergseth MF, Ørn S (2020) Artifact correction in short-term HRV during strenuous physical exercise. Sensors (Basel). 10.3390/s20216372 PMC7664660 33171676

[39] Burma JS, Griffiths JK, Lapointe AP, Oni IK, Soroush A, Carere J et al (2024) Heart rate variability and pulse rate variability: do anatomical location and sampling rate matter? Sensors. 10.3390/s24072048 PMC11013825 38610260

[40] Pelaez-Coca MD, Hernando A, Lazaro J, Gil E (2022) Impact of the PPG sampling rate in the pulse rate variability indices evaluating several fiducial points in different pulse waveforms. IEEE J Biomed Health Inf 26(2):539–549. 10.1109/JBHI.2021.3099208 34310329

[41] Mather JD, Hayes LD, Mair JL, Sculthorpe NF (2024) Validity of resting heart rate derived from contact-based smartphone photoplethysmography compared with electrocardiography: a scoping review and checklist for optimal acquisition and reporting. Front Digit Health 6:1326511. 10.3389/fdgth.2024.1326511 PMC10937558 38486919

[42] Reali P, Lolatto R, Coelli S, Tartaglia G, Bianchi AM (2022) Information retrieval from photoplethysmographic sensors: a comprehensive comparison of practical interpolation and breath-extraction techniques at different sampling rates. Sensors. 10.3390/s22041428 PMC8877143 35214329

[43] Malliani A, Pagani M, Lombardi F, Cerutti S (1991) Cardiovascular neural regulation explored in the frequency domain. Circulation 84(2):482–492. 10.1161/01.cir.84.2.482 1860193

[44] Eckberg DL (1997) Sympathovagal balance: a critical appraisal. Circulation 96(9):3224–3232. 10.1161/01.cir.96.9.3224 9386196

[45] Beauchaine TP, Thayer JF (2015) Heart rate variability as a transdiagnostic biomarker of psychopathology. Int J Psychophysiol 98(2 Pt 2):338–350. 10.1016/j.ijpsycho.2015.08.004 26272488

[46] Tomasi J, Zai CC, Pouget JG, Tiwari AK, Kennedy JL (2024) Heart rate variability: evaluating a potential biomarker of anxiety disorders. Psychophysiology 61(2):e14481. 10.1111/psyp.14481 37990619

[47] Villar de Araujo T, Rüesch A, Bankwitz A, Rufer M, Kleim B, Olbrich S (2023) Autism spectrum disorders in adults and the autonomic nervous system: heart rate variability markers in the diagnostic procedure. J Psychiatr Res 164:235–242. 10.1016/j.jpsychires.2023.06.006 37385002

[48] Gullett N, Zajkowska Z, Walsh A, Harper R, Mondelli V (2023) Heart rate variability (HRV) as a way to understand associations between the autonomic nervous system (ANS) and affective states: a critical review of the literature. Int J Psychophysiol 192:35–42. 10.1016/j.ijpsycho.2023.08.001 37543289

[49] Mulcahy JS, Larsson DEO, Garfinkel SN, Critchley HD (2019) Heart rate variability as a biomarker in health and affective disorders: a perspective on neuroimaging studies. Neuroimage 202:116072. 10.1016/j.neuroimage.2019.116072 31386920

[50] Rüesch A, Villar de Araujo T, Bankwitz A, Hörmann C, Adank A, Ip CT et al (2023) A recent suicide attempt and the heartbeat: Electrophysiological findings from a trans-diagnostic cohort of patients and healthy controls. J Psychiatr Res 157:257–263. 10.1016/j.jpsychires.2022.11.020 36516500

[51] Olbrich S, Tränkner A, Surova G, Gevirtz R, Gordon E, Hegerl U et al (2016) CNS- and ANS-arousal predict response to antidepressant medication: findings from the randomized iSPOT-D study. J Psychiatr Res 73:108–15. 10.1016/j.jpsychires.2015.12.001 26714202

[52] Meyer T, Brunovsky M, Horacek J, Novak T, Andrashko V, Seifritz E et al (2021) Predictive value of heart rate in treatment of major depression with ketamine in two controlled trials. Clin Neurophysiol 132(6):1339–46. 10.1016/j.clinph.2021.01.030 33888426

[53] Quigley KS, Gianaros PJ, Norman GJ, Jennings JR, Berntson GG, de Geus EJC (2024) Publication guidelines for human heart rate and heart rate variability studies in psychophysiology-Part 1: physiological underpinnings and foundations of measurement. Psychophysiology 61(9):e14604. 10.1111/psyp.14604 PMC11539922 38873876

[54] Fine J, Branan KL, Rodriguez AJ, Boonya-Ananta T, Ajmal, Ramella-Roman JC et al (2021) Sources of inaccuracy in photoplethysmography for continuous cardiovascular monitoring. Biosens (Basel). 10.3390/bios11040126 PMC8073123 33923469

[55] Goessl VC, Curtiss JE, Hofmann SG (2017) The effect of heart rate variability biofeedback training on stress and anxiety: a meta-analysis. Psychol Med 47(15):2578–2586. 10.1017/S0033291717001003 28478782

[56] Laborde S, Allen MS, Borges U, Dosseville F, Hosang TJ, Iskra M et al (2022) Effects of voluntary slow breathing on heart rate and heart rate variability: a systematic review and a meta-analysis. Neurosci Biobehav Rev 138:104711. 10.1016/j.neubiorev.2022.104711 35623448

[57] Steffen PR, Bartlett D, Channell RM, Jackman K, Cressman M, Bills J et al (2021) Integrating breathing techniques into psychotherapy to improve HRV: which approach is best? Front Psychol 12:624254. 10.3389/fpsyg.2021.624254 PMC7917055 33658964

